# Multimorbidity in Atherosclerotic Cardiovascular Disease and Its Associations With Adverse Cardiovascular Events and Healthcare Costs: A Real-World Evidence Study

**DOI:** 10.36469/001c.94710

**Published:** 2024-03-22

**Authors:** Dingwei Dai, Joaquim Fernandes, Xiaowu Sun, Laura Lupton, Vaughn W. Payne, Alexandra Berk

**Affiliations:** 1 CVS Health, Woonsocket, Rhode Island, USA

**Keywords:** atherosclerotic cardiovascular disease, comorbidity, multimorbidity, adverse cardiovascular events, secondary prevention, healthcare costs, real-world evidence

## Abstract

**Background:** Atherosclerotic cardiovascular disease (ASCVD) remains the leading cause of mortality and disability in the United States and worldwide.

**Objective:** To assess the multimorbidity burden and its associations with adverse cardiovascular events (ACE) and healthcare costs among patients with ASCVD.

**Methods:** This is a retrospective observational cohort study using Aetna claims database. Patients with ASCVD were identified during the study period (1/1/2018–10/31/2021). The earliest ASCVD diagnosis date was identified as the index date. Qualified patients were ≥18 years of age and had ≥12 months of health plan enrollment before and after the index date. Comorbid conditions were assessed using all data available within 12 months prior to and including the index date. Association rule mining was applied to identify comorbid condition combinations. ACEs and healthcare costs were assessed using all data within 12 months after the index date. Multivariable generalized linear models were performed to examine the associations between multimorbidity and ACEs and healthcare costs.

**Results:** Of 223 923 patients with ASCVD (mean [SD] age, 73.6 [10.7] years; 42.2% female), 98.5% had ≥2, and 80.2% had ≥5 comorbid conditions. The most common comorbid condition dyad was hypertension-hyperlipidemia (78.7%). The most common triad was hypertension–hyperlipidemia–pain disorders (61.1%). The most common quartet was hypertension–hyperlipidemia–pain disorders–diabetes (30.2%). The most common quintet was hypertension–hyperlipidemia–pain disorders–diabetes–obesity (16%). The most common sextet was hypertension–hyperlipidemia–pain disorders–diabetes–obesity–osteoarthritis (7.6%). The mean [SD] number of comorbid conditions was 7.1 [3.2]. The multimorbidity burden tended to increase in older age groups and was comparatively higher in females and in those with higher social vulnerability. The increased number of comorbid conditions was significantly associated with increased ACEs and increased healthcare costs.

**Discussion:** Extremely prevalent multimorbidity should be considered in the context of clinical decision-making to optimize secondary prevention of ASCVD.

**Conclusions:** Multimorbidity was extremely prevalent among patients with ASCVD. Multimorbidity patterns varied considerably across ASCVD patients and by age, gender, and social vulnerability status. Multimorbidity was strongly associated with ACEs and healthcare costs.

## BACKGROUND

Atherosclerotic cardiovascular (CV) disease (ASCVD) remains the leading cause of morbidity and mortality in the United States (US) and worldwide, accounting for substantial suffering and healthcare-related expenditures.^1–3^ Most patients with ASCVD have a high burden of CV and non-CV comorbid conditions and multimorbidity.^4^ In a 2018 claims data analysis from the US Centers for Medicare & Medicaid Services (CMS), 22% of women and 32% of men in the US Medicare population had ischemic heart disease, and more than 70% had at least 2 defined chronic conditions.^2,5^ In another CMS report, over 50% of patients with heart failure (HF) or stroke had at least 5 defined chronic conditions.^6^

ASCVD increases with age, as do the number and variety of disease and other chronic conditions,^2,7^ with the burden and impact of multimorbidity usually becoming more severe over time.^7^ Healthcare providers often manage ASCVD patients with multimorbidity, interrelating pathophysiology, such as coronary artery disease (CAD), diabetes mellitus (DM), and hypertension. ASCVD patients with multimorbidity require multiple therapies, including those targeting ASCVD itself and others to treat comorbid conditions or mitigate their associated risk factors. Current clinical practice guidelines provide disease-specific management strategies; however, such single-disease guidelines may no longer be appropriate when care is complicated by multimorbidity. The American College of Cardiology (ACC) recently developed a framework for pragmatic, patient-centered care for ASCVD patients with multimorbidity.^2^

While numerous data exist on the prevalence of individual chronic conditions among patients with ASCVD,^4–7^ the prevalence of specific comorbid condition combinations is lacking. Real-world evidence on the prevalence of multimorbidity and its impact in ASCVD is limited. Clear recognition and characterization of comorbidity and multimorbidity patterns are crucial to understand a patient’s overall health and ASCVD risk, and to mitigate disease progression, prevent adverse CV events (ACEs), and reduce healthcare costs. Therefore, this study aims to evaluate the prevalence of individual comorbid conditions and multimorbidity among adult patients with ASCVD and examine the associations between multimorbidity and ACEs and healthcare costs using a large US healthcare claims database.

## METHODS

### Study Design and Data Source

This is a retrospective observational cohort study of patients with ASCVD using a large database of U.S. administrative claims data from January 1, 2018, to December 31, 2021 (**Supplementary Figure S1**). The claims database included over 20 million commercial fully medical insured and Medicare Advantage health plan beneficiaries, and contained patient demographics (age, gender, race/ethnicity, geographic region, and residential rural/urban area), health plan enrollment, medical claims for both inpatient and outpatient medical services, pharmacy claims, and laboratory data for commercial fully insured and Medicare Advantage members.^8,9^ The study protocol was approved by the Sterling institutional review board (IRB ID: 9472-DDai), which waived the need for patient informed consent because the study was deemed to pose minimal risk. This study followed the Strengthening the Reporting of Observational Studies in Epidemiology (STROBE) guidelines.^10^

### Study Population

The definition of ASCVD was based on the ACC/American Heart Association (AHA) definition and included acute coronary syndrome (ACS), history of myocardial infarction (MI), stable or unstable angina, coronary or other arterial revascularization, stroke, transient ischemic attack (TIA), peripheral artery disease (PAD), or aortic aneurysm, all of atherosclerotic origin.^11^ Patients with at least 1 ASCVD-related *International Classification of Diseases, Tenth Revision, Clinical Modification* (ICD-10-CM) diagnosis or procedure codes for any these conditions in medical claims data were identified as having ASCVD.^12^ The first observed ASCVD diagnosis date was defined as an index date within the index period (**Supplementary Figure S1**). Patients with ASCVD were eligible for inclusion in the study if they were at least 18 years of age and had a fully insured commercial health plan or Medicare Advantage coverage with medical and pharmacy health insurance benefits for at least 12 months prior to the index date (baseline period) and 12 months after the index date (follow-up period).

### Baseline Sociodemographic Characteristics and Comorbidities

The patient sociodemographic characteristics evaluated included age at index date, gender, geographic region, rural, suburban, or urban residence, median household income, and social vulnerability index (SVI). Household income was estimated by linking 2010 US Census data to the cohort data using zip code. The Centers for Disease Control and Prevention (CDC)’s SVI was developed by the Agency for Toxic Substance and Disease Registry’s (ATSDR) Geospatial Research, Analysis and Services Program to provide a composite measure of community susceptibility to adversities in the face of disasters, including disease outbreaks.^13,14^ The CDC/ATSDR SVI ranks each census tract on 16 social factors, including poverty, lack of vehicle access, crowded housing, racial minority and limited English speaking, and groups these relative factors into 4 related themes: socioeconomic status, household characteristics and disability, minority status and language, and housing and transportation. The overall SVI is calculated by adding up the values of the 4 themes and converting the summated score into a percentile rank. The individual theme and overall scores range from 0 to 1, with higher values indicating greater social vulnerability. More information about the SVI methodologies and database can be found at the CDC/ATSDR SVI homepage.^14^ For this analysis, the CDC/ATSDR SVI 2020 database was used.^14^

### Assessment of Comorbidity and Multimorbidity

The 42 most common chronic conditions in the Aetna claims data were included and identified using previously described methods based on ICD-10-CM codes.^8,15^ The list of each chronic condition, its acronym or abbreviation, and the corresponding ICD-10-CM codes used in this study are reported in **Supplementary Table S1**. We included 18 of the 20 comorbid conditions recommended by the US Department of Health and Human Services for studying multimorbidity because they are chronic, prevalent, and potentially amenable to intervention.^16,17^ We excluded autism and CAD because autism is rare in the population, and CAD is the index condition in this study. Multimorbidity was defined as the presence of at least 2 comorbid conditions within a patient.^15–19^ The number of comorbid conditions was a count of all specified comorbid conditions in each patient. Up to 6 comorbid condition combinations were identified. Presence of comorbid conditions was assessed using all available data within 12 months prior to and including the index date. The Charlson Comorbidity Index (CCI), a validated algorithm that characterizes comorbidity burden and predicts risk of mortality and increased healthcare costs, was also calculated.^20,21^

### Adverse Cardiovascular Events

Clinical adverse CV events (ACEs) included ACS, acute MI (AMI), unstable angina, stroke, PAD, coronary revascularization, HF, hospitalization for HF, and major adverse CV events (MACE) defined as a composite of AMI and stroke. The list of each ACE and the corresponding ICD-10-CM codes used in this study is reported in **Supplementary Table S2**. Hospitalization for HF was identified as having hospitalization with primary diagnosis of HF during the follow-up period.

### Healthcare Costs

Healthcare costs were defined as the sum of all all-cause costs for any emergency department visits, outpatient services, inpatient admission, laboratory, radiology, operating room, supplies, pharmacy, and other ancillary services over the 12-month follow-up period.^8,9^ All healthcare costs were inflated to 2021 US dollars using the Consumer Price Indices for the medical care services component.^22,23^

### Statistical Analysis

Means (±SD) or medians (interquartile range) were reported for continuous variables, and frequencies (%) were reported for categorical variables. To compare differences between age groups (<65, ≥65 years old) and sex, statistical significance was assessed with the Student’s *t*-test, Wilcoxon rank-sum test, or Kruskal-Wallis test for continuous variables, and the Pearson chi-square test for categorical variables.

Association rule mining was used to identify the most prevalent combinations of 42 comorbid conditions, the algorithm and method were described in our previous study.^8^ To describe the patterns of comorbidity and multimorbidity, we calculated the number of discrete comorbid conditions for each patient and computed prevalence rates of individual 2 to 5 comorbid condition combinations. Analyses were conducted for the overall population, as well as by age groups and gender. Wilcoxon rank-sum tests were used to compare the mean numbers of comorbid conditions between age groups and gender. Pearson chi-square tests were used to compare the rates of individual comorbid conditions or combinations of 2 to 6 comorbid conditions between age groups and gender. The Jonckheere-Terpstra test, a rank-based trend test, was used to assess the correlations between number of comorbid conditions and quartiles of household incomes and SVI.

To assess the association between the number of comorbid conditions and each ACE (including ACS, AMI, unstable angina, stroke, PAD, coronary revascularization, HF, hospitalization for HF, and MACE) and total healthcare costs, we also performed multivariable generalized linear regression models to calculate adjusted odds ratios (AOR) and cost ratios (ACR) with corresponding 95% confidence interval (CI) after adjusting for other potential contributors: gender, age, median household income, SVI, geographic region, rural-urban residence, and type of health insurance (commercial insurance or Medicare Advantage). Logistic regression models were used for ACEs (binary outcome). The generalized linear model with gamma distribution and log link function for maximum-likelihood estimation was used for total healthcare cost.^8,9^

All statistical analyses were conducted using SAS version 9.4 statistical analysis software and SAS Enterprise Miner version 15.1 (SAS Institute Inc.). All *P* values were 2-sided, with a level of .05 considered to indicate statistically significance.

## RESULTS

### Study Population

We identified a total of 223 923 eligible adult patients with ASCVD (**Supplementary Figure S2**). Mean (SD) age was 73.6 (10.7) years, 57.8% were male, 82.8% were at least age 65 years, 49.8% lived in the southern United States, 46.8% lived in rural areas, median household income was $56,718, and median SVI was 0.41. Medicare patients made up 89.3% of the study population; the remaining 10.7% were patients with commercial health insurance (**Table 1**). Among 223 923 patients with ASCVD, mean (SD) CCI was 3.28 (2.45). CCI was significantly higher in those aged 65 years and over than in those under 65 (3.39 (2.45) vs 2.75 (2.42), *P* < .0001), and higher in females than males (3.34 (2.41) vs 3.24 (2.48), *P* < .0001).

**Table 1.** Sociodemographic Characteristics of Patients with ASCVD and Their Associations With Number of Comorbid Conditions

**Characteristics**	**Overall (N=223 923), %**	**No. of Comorbidities, Mean (95% CI)**	***P* Value**
Age (years), mean (SD)	73.58 (10.73)	7.09 (7.08-7.11)	
Age category, years			<.0001
18-34	610 (0.27)	4.56 (4.22-4.70)	
35-44	2103 (0.94)	5.26 (5.12-5.39)	
45-54	8829 (3.94)	5.97 (5.90-6.04)	
55-64	26 887 (12.01)	6.64 (6.60-6.83)	
65-74	76 200 (34.03)	6.86 (6.84-6.88)	
75-84	75 860 (33.88)	7.41 (7.37-7.42)	
≥85	33 434 (14.93)	7.77 (7.74-7.81)	
Sex			<.0001
Female	94 557 (42.23)	7.75 (7.72-7.77)	
Male	129 364 (57.77)	6.62 (6.60-6.64)	
Race			<.0001
Black	20 807 (9.29)	7.40 (7.35-7.44)	
White	141 025 (62.98)	7.06 (7.04-7.08)	
Hispanic	2907 (1.30)	6.76 (6.64-6.88)	
Asian	2716 (1.21)	5.74 (5.64-5.85)	
≥2 races	1977 (0.88)	6.29 (6.15-6.42)	
Other	3678 (1.64)	6.35 (6.26-6.46)	
Unknown	50 813 (22.69)	7.24 (7.20-7.27)	
Geographic regions			<.0001
Midwest	71 983 (32.15)	7.14 (7.11-7.16)	
Northeast	50 662 (22.63)	7.30 (7.27-7.33)	
South	89 032 (39.76)	7.06 (7.03-7.08)	
West	12 246 (5.47)	6.26 (6.20-6.32)	
Urban-rural area			<.0001
Urban	54 310 (24.25)	6.96 (6.93-6.99)	
Suburban	64 820 (28.95)	7.07 (7.04-7.09)	
Rural	104 793 (46.80)	7.18 (7.16-7.20)	
Payers			<.0001
Commercial insurance	23 920 (10.68)	5,19 (5.15-5.23)	
Medicare Advantage	200 003 (89.32)	7.32 (7.30-7.34)	
Median household income (US $)			
Lowest quartile (<$45 355)	56 005 (25.01)	7.49 (7.46-7.52)	<.0001
2nd quartile (45 355−56 747)	56 436 (25.20)	7.23 (7.20-7.26)	
3rd quartile (56 748−73 260)	55 526 (24.80)	7.06 (7.03-7.09)	
Highest quartile (>$73 260)	55 956 (24.99)	6.59 (6.56-6.62)	
Social Vulnerability Index			<.0001
Lowest quartile, least vulnerability (<0.201)	55 986 (25.00)	6.85 (6.82-6.88)	
2nd quartile (0.202-0.407)	56 582 (25.27)	7.02 (6.99-7.05)	
3rd quartile (0.408-0.681)	55 391 (24.74)	7.23 (7.19-7.25)	
Highest quartile, highest vulnerability (>0.681)	55 964 (24.99)	7.29 (7.26-7.31)	

Abbreviations: ASCVD, atherosclerotic cardiovascular disease; CI, confidence interval; SVI, Social Vulnerability Index.

### Comorbidity and Multimorbidity in ASCVD

**Table 2** presents the prevalence of the top 30 most common chronic conditions in patients with ASCVD. The most common comorbid conditions include hypertension (88.8%), hyperlipidemia (86%), pain disorders (76.3%), DM (41.9%), and obesity (38%). The prevalence of individual comorbid conditions in ASCVD varied by age groups and gender (**Table 2**). **Table 3** shows the prevalence of the 10 most prevalent comorbid condition combinations from 2 to 6 comorbid condition combinations, overall and by age group and gender. The most common comorbid condition dyads were hypertension-hyperlipidemia (78.7%) and hypertension–pain disorders (68.7%). The most common triads were hypertension–hyperlipidemia–pain disorders (61.1%) and hypertension-hyperlipidemia-DM (37.1%). The most common quartets were hypertension–hyperlipidemia–pain disorders–DM (30.2%) and hypertension–hyperlipidemia–pain disorders–obesity (26.9%). The most common quintets were hypertension–hyperlipidemia–pain disorders–DM–obesity (16%) and hypertension–hyperlipidemia–pain disorders–DM–osteoarthritis (13.2%). The most common sextets were hypertension–hyperlipidemia–pain disorders–DM–obesity–osteoarthritis (7.6%) and hypertension–hyperlipidemia–pain disorders–DM–obesity–fatigue/sleep-related disorders (7.2%).

**Table 2.** Prevalence of the 30 Most Common Comorbid Conditions Among Patients With ASCVD by Age Group and Gender

**Comorbid Conditions**	**Overall (n=223 923)**	**Age (Years)**			**Gender**		
		**<65 (n = 38 429)**	**≥65 (n = 185 494)**	***P* Value**	**Female (n = 94 557)**	**Male (n = 129 366)**	***P* Value**
Hypertension	88.84	78.84	90.91	<.0001	89.00	88.72	.0357
Hyperlipidemia	85.95	76.97	87.81	<.0001	84.11	87.29	<.0001
Pain disorders	76.26	76.87	76.13	.0021	81.89	72.15	<.0001
Diabetes mellitus	41.92	40.28	42.26	<.0001	40.96	42.62	<.0001
Obesity	37.97	42.52	37.03	<.0001	38.76	37.39	<.0001
Fatigue and sleep-related disorders	37.18	34.35	37.76	<.0001	42.71	33.13	<.0001
Osteoarthritis	35.18	23.40	37.62	<.0001	42.44	29.88	<.0001
Heart failure and nonischemic heart disease	24.67	19.12	25.81	<.0001	24.78	24.58	0.2815
Chronic thyroid disorders	23.53	17.11	24.85	<.0001	33.31	16.37	<.0001
Chronic kidney disease	23.50	13.54	25.57	<.0001	23.32	23.64	.0772
Chronic obstructive pulmonary disease	23.24	18.20	24.29	<.0001	24.79	22.11	<.0001
Atrial fibrillation	22.92	10.70	25.45	<.0001	21.22	24.16	<.0001
Cigarette smoking	19.38	22.64	18.71	<.0001	17.52	20.74	<.0001
Anxiety	19.03	23.92	18.02	<.0001	26.87	13.30	<.0001
Depression	18.37	21.26	17.77	<.0001	25.22	13.36	<.0001
Substance use disorders	14.67	25.18	12.49	<.0001	13.38	15.61	<.0001
Cancer (malignancy)	14.21	8.06	15.48	<.0001	12.05	15.79	<.0001
Diverticular disease	11.73	8.00	12.50	<.0001	12.42	11.22	<.0001
Ventricular arrhythmias	10.47	8.49	10.88	<.0001	9.47	11.20	<.0001
Osteoporosis	9.91	3.72	11.19	<.0001	19.76	2.70	<.0001
Asthma	8.96	11.32	8.47	<.0001	12.44	6.41	<.0001
Iron deficiency anemia	8.86	6.99	9.25	<.0001	10.96	7.33	<.0001
Schizophrenia	7.92	2.65	9.01	<.0001	10.08	6.33	<.0001
Rheumatoid arthritis/collagen vascular disease	7.48	7.76	7.42	.0191	10.59	5.20	<.0001
Alzheimer’s disease/dementia	7.46	1.51	8.69	<.0001	9.47	5.98	<.0001
Liver disease	6.98	9.95	6.38	<.0001	7.27	6.79	<.0001
Aortic valve stenosis	6.28	1.90	7.19	<.0001	6.15	6.38	0.0281
Kidney stones	5.16	5.01	5.20	.1335	3.85	6.13	<.0001
Inflammatory bowel disease	4.22	4.42	4.18	.0341	5.32	3.42	<.0001
Other nontoxic goiter	4.08	4.10	4.08	.8748	6.44	2.36	<.0001

Abbreviations: ASCVD, atherosclerotic cardiovascular disease.

**Table 3.** Prevalence of the 10 Most Common Comorbid Condition Combinations from 2 to 6 Conditions in Combination Among Patients With ASCVD by Age Group and Gender

**Comorbid Condition Combinations**	**Overall** **(n = 223 923)**	**Age (Years)**			**Gender**		
		**<65 (n = 38 429)**	**≥65 (n = 185 494)**	***P* Value**	**Female (n = 94 557)**	**Male (n = 129 366)**	***P* Value**
Dyads							
HLD-HTN	78.72	65.46	81.47	<.0001	77.35	79.72	<.0001
HTN-PAI	68.67	61.95	70.06	<.0001	73.59	65.08	<.0001
HLD-PAI	66.18	59.88	67.48	<.0001	69.53	63.73	<.0001
DM-HTN	40.01	37.03	40.61	<.0001	39.27	40.53	<.0001
DM-HLD	38.56	35.46	39.20	<.0001	37.38	39.43	<.0001
HTN-OBE	35.66	37.61	35.25	<.0001	36.32	35.17	<.0001
HLD-OBE	34.40	35.61	34.14	<.0001	34.45	34.35	.6342
FSR-HTN	33.85	28.19	35.02	<.0001	38.79	30.24	<.0001
DM—PAI	33.76	33.01	33.91	.0006	34.89	32.93	<.0001
HTN-OST	32.46	20.25	34.99	<.0001	39.20	27.53	<.0001
Triads							
HLD-HTN-PAI	61.12	51.68	63.08	<.0001	64.27	58.82	<.0001
HLD-HTN-DM	37.10	33.19	37.91	<.0001	36.11	37.82	<.0001
HLD-HTN-OBE	32.71	32.55	32.75	.4433	32.78	32.67	.5912
HYP-PAI-DM	32.41	30.68	32.77	<.0001	33.58	31.55	<.0001
DM-HLD-PAI	31.19	29.27	31.59	<.0001	31.98	30.62	<.0001
HLD-HTN-FSR	30.26	23.75	31.61	<.0001	34.08	27.46	<.0001
HTN-OBE-PAI	29.36	31.56	28.91	<.0001	31.55	27.76	<.0001
HTN-PAI-FSR	29.29	24.93	30.20	<.0001	34.72	25.33	<.0001
HLD-HTN-OST	29.16	17.33	31.62	<.0001	34.62	25.18	<.0001
HTN-PAI-OST	28.76	18.67	30.85	<.0001	35.58	23.77	<.0001
Quartets							
HLD-HTN-PAI-DM	30.16	27.63	30.68	<.0001	31.00	29.54	<.0001
HLD-HTN-PAI-OBE	26.94	27.34	26.86	.0525	28.49	25.81	<.0001
HLD-HTN-PAI-FSR	26.25	21.06	27.33	<.0001	30.60	23.08	<.0001
HLD-HTN-PAI-OST	25.87	15.96	27.93	<.0001	31.48	21.78	<.0001
HLD-HTN-DIA-OBE	18.79	19.69	18.62	<.0001	19.00	18.65	.0400
HLD-HTN-PAI-CHF	17.83	12.74	18.88	<.0001	18.60	17.27	<.0001
HLD-HTN-PAI-CKD	17.21	9.70	18.76	<.0001	17.72	16.84	<.0001
HTN-PAI-DM-OBE	16.93	18.67	16.57	<.0001	18.03	16.13	<.0001
DM-HLD-PAI-OBE	16.36	17.77	16.07	<.0001	17.26	15.71	<.0001
HLD-HTN-PAI-COP	16.29	12.33	17.11	<.0001	17.88	15.13	<.0001
Quintets							
HLD-HTN-PAI-DM-OBE	16.01	17.13	15.78	<.0001	16.89	15.36	<.0001
HLD-HTN-PAI-DM-FSR	13.19	11.64	13.52	<.0001	14.81	12.02	<.0001
HLD-HTN-PAI-OBE-OST	12.98	10.09	13.59	<.0001	15.76	10.96	<.0001
HLD-HTN-PAI-DM-OST	12.76	9.11	13.52	<.0001	15.22	10.97	<.0001
HLD-HTN-PAI-FSR-OST	12.55	7.86	15.53	<.0001	16.44	9.72	<.0001
HLD-HTN-PAI-OBE-FSR	11.99	12.10	11.97	.4729	13.87	10.61	<.0001
HLD-HTN-PAI-DM-CKD	10.72	7.22	11.45	<.0001	10.82	10.65	.2131
HLD-HTN-PAI-DM-CHF	10.28	8.41	10.66	<.0001	10.40	10.19	.1089
HLD-HTN-PAI-FSR-CHF	9.10	6.19	9.71	<.0001	10.29	8.23	<.0001
HLD-HTN-PAI-OBE-CHF	9.04	7.92	9.27	<.0001	9.52	8.69	<.0001
Sextets							
HLD-HTN-PAI-DM-OBE-OST	7.61	6.55	7.83	<.0001	9.24	6.43	<.0001
HLD-HTN-PAI-DM-OBE-FSR	7.23	7.72	7.13	<.0001	8.24	6.49	<.0001
HLD-HTN-PAI-DM-OST-FSR	6.30	4.59	6.65	<.0001	7.99	5.06	<.0001
HLD-HTN-PAI-DM-OBE-CHF	6.04	5.80	6.09	.0305	6.29	5.86	<.0001
HLD-HTN-PAI-DM-OBE-CKD	5.98	4.85	6.21	<.0001	6.29	5.74	<.0001
HLD-HTN-PAI-DM-FSR-CHF	5.29	4.18	5.52	<.0001	5.76	4.94	<.0001
HLD-HTN-PAI-DM-FSR-CKD	5.21	3.48	5.57	<.0001	5.63	4.91	<.0001
HLD-HTN-PAI-DM-CKD-CHF	5.20	3.65	5.53	<.0001	5.28	5.15	.1981
HLD-HTN-PAI-DM-OST-CKD	4.83	2.54	5.30	<.0001	5.63	4.24	<.0001
HLD-HTN-PAI-DM-OBE-COP	4.80	5.02	4.75	.0230	5.17	4.52	<.0001

Abbreviations: ASCVD, atherosclerotic cardiovascular disease; CHF, heart failure and non-ischemic heart disease; CKD, chronic kidney disease; COP, chronic obstructive pulmonary disease; DM, diabetes mellitus; FSR, fatigue and sleep-related disorders; HLD, hyperlipidemia; HTN, hypertension; OBE, obesity; OST, osteoarthritis; PAI, pain disorders.

**Figure 1.**
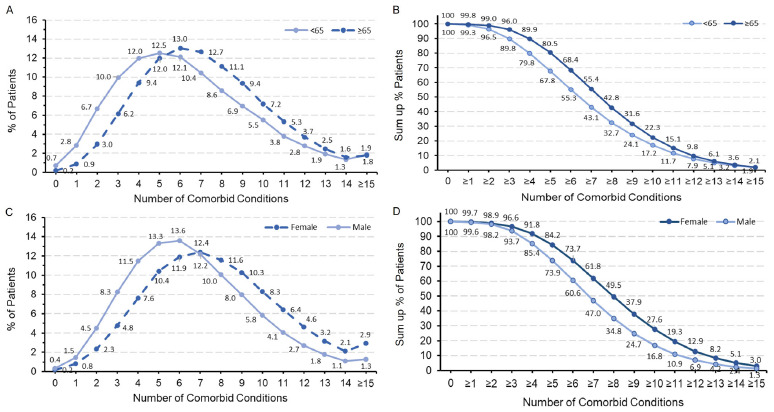
Proportion of ASCVD Patients With the Indicated Number of Comorbid Conditions and Summed Proportions of Multimorbidity With the Indicated Number of Comorbid Conditions (**A**) Proportion of ASCVD patients with the indicated number of comorbid conditions by age group. (**B**) Summed proportions of multimorbidity with the indicated number of comorbid conditions by age group. (**C**) Proportion of ASCVD patients with the indicated number of comorbid conditions by gender. (**D**) Summed proportions of multimorbidity with the indicated number of comorbid conditions by age and gender. Abbreviation: ASCVD, atherosclerotic cardiovascular disease.

Based on these 42 comorbid conditions (**Supplementary Table S1**), the number of comorbid conditions among patients with ASCVD ranged from 0 to 21 in this cohort; median (interquartile range) values were 7 (5-9) for overall, 6 (4-8) for age <65 years, 7 (5-9) for age ≥65, 7 (5-10) for females, and 6 (4-8) for males. Overall, 98.5% of patients with ASCVD had ≥2 comorbid conditions, 80.2% had ≥5 comorbid conditions, and 25.7% had ≥10 comorbid conditions. The proportions and summed proportions of multimorbidity in patients with ASCVD stratified by age groups and gender are shown in **Figure 1**. The most common numbers of comorbid conditions were 5 in those age <65 (12.5%), 6 in those age ≥65 (13.0%, **Figure 1A**), 6 in male patients (13.6%), and 7 in female patients (12.4%, **Figure 1C**). The proportions of multimorbidity with 6 to 12 comorbid conditions were significantly higher in age ≥65 than age <65 (all *P* < .0001, **Figure 1A**). The proportions of multimorbidity with 8 and more comorbid conditions were significantly higher in females than in males (all *P* < .0001, **Figure 1C)**. The summed proportions of multimorbidity with 2 or more comorbid conditions were significantly higher in age ≥65 than age <65 (all *P* < .0001, **Figure 1B**), and higher in females than males (all *P* < .0001, **Figure 1D**).

### Association of Sociodemographic Characteristics With Multimorbidity

**Table 1** shows mean numbers of comorbid conditions in ASCVD by patient sociodemographic characteristics. The number of comorbid conditions among patients with ASCVD increased with age in the study population (Jonckheere-Terpstra test, *z* = 73.71, *P* < .0001), in females (*z* = 35.03, *P* < .0001) and males (*z* = 58.75, *P* < .0001). The mean number of comorbid conditions in females was higher than in males (7.8 vs 6.6, *P* < .0001). The number of comorbid conditions varied by race with the highest number in black patients (7.4) and lowest number in Asian patients (5.7), by geographic regions with the highest number in the Midwest (7.3) and lowest number in the West (6.3), by urban-rural residence area with the highest number in rural (7.2) and lowest number in urban areas (7), all *P* < .0001. Patients on a Medicare Advantage plan had higher comorbidity burdens than patients with commercial health insurance (7.3 vs 5.2, *P* < .0001). The number of comorbid conditions among patients with ASCVD decreased with increased household income (*z* = -48.69, *P* < .⁠0001) and lower social vulnerability (*z* = 25.84, *P* < .0001, **Table 1**).

### Association of Multimorbidity With Adverse Cardiovascular Events

**Figure 2.**
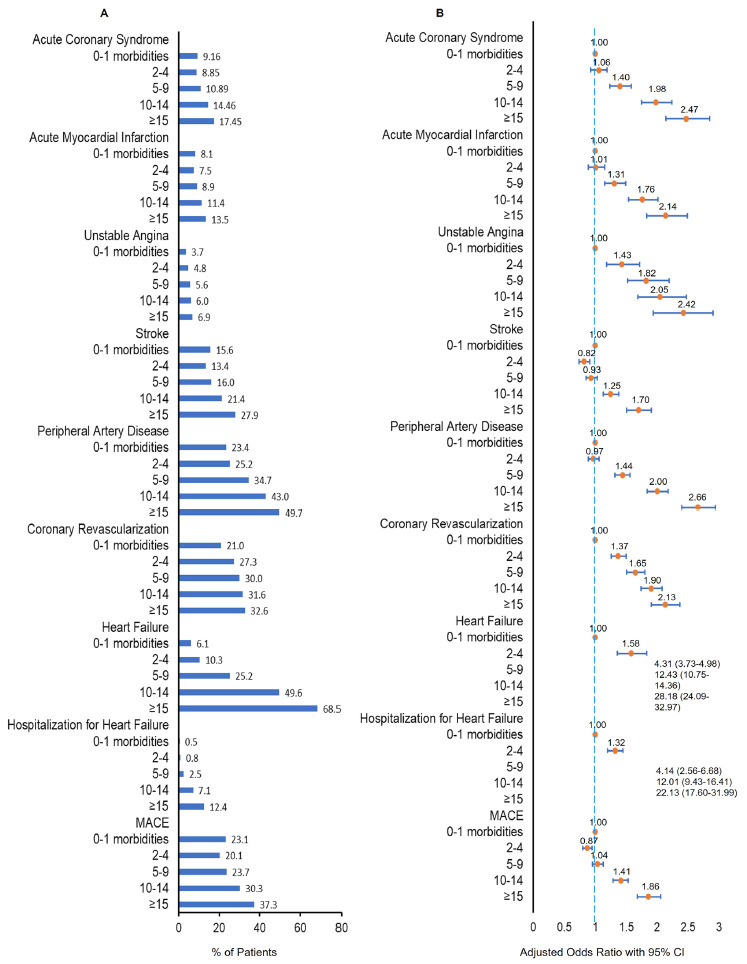
Prevalence of Adverse Cardiovascular Events (ACEs) by Number of Comorbid Conditions Group (**A**) and Adjusted Odds Ratio of Comorbid Conditions Group for ACEs (**B**) (**A**) Bar chart of prevalence of ACEs during the 12-month follow-up period by number of comorbid conditions group among patients with ASCVD. (**B**) Forest plot of multivariable adjusted odds ratios (AOR) of number of comorbid conditions group for ACEs. Adjusted variables included age, gender, geographic region, rural or urban residence, household income, Social Vulnerability Index, and type of health insurance. The v*ertical dashed blue line* represents an AOR of 1 as the reference line, which is associated with equal odds for all number of comorbid conditions groups. For each AOR displayed, the *orange circle symbol* depicts the AOR, and the *horizontal blue line* represents the 95% CI. Lines that do not cross the reference line are statistically significant. Abbreviations: ACEs, adverse cardiovascular events; ASCVD, atherosclerotic cardiovascular disease; CI, confidence interval; MACE, major adverse cardiovascular events.

**Supplementary Figure S3** shows that the proportion of patients with ACEs (including ACS, AMI, unstable angina, stroke, PAD, coronary revascularization, HF, hospitalization for HF, and MACE) was elevated with increased numbers of comorbid conditions in patients with ASCVD. After adjusting for other covariables (including age, gender, geographic region, rural or urban residence, household income, SVI, and type of health insurance), there was a 7.5% odds increase for ACS among ASCVD patients with each additional comorbid condition (AOR [95% CI]: 1.075 [1.07-1.08]), a 6.5% odds increase for AMI (1.065% [1.06-1.07]), a 4.6% odds increase for unstable angina (1.046% [1.04-1.05]), a 5.5% odds increase for stroke (1.055% [1.05-1.06]), an 8.8% odds increase for PAD (1.088% [1.085-1.092]), a 4% odds increase for coronary revascularization (1.04% [1.037-1.43]), a 28.2% odds increase for HF (1.282% [1.278-1.286]), a 24.7% odds increase for hospitalization for HF (1.247% [1.239-1.256]), and a 6% odds increase for MACE (1.060% [1.057-1.063]), all *P* < .0001. We also found similar patterns by categorizing the number of comorbid conditions into 5 multimorbidity burden groups. In multivariable analyses (**Figure 2**), compared with patients without multimorbidity (0-1 morbidities), higher multimorbidity burden was associated with higher prevalence of ACEs in patients with ASCVD (all *P* < .⁠001).

### Association of Multimorbidity with Healthcare Cost

**Figure 3.**
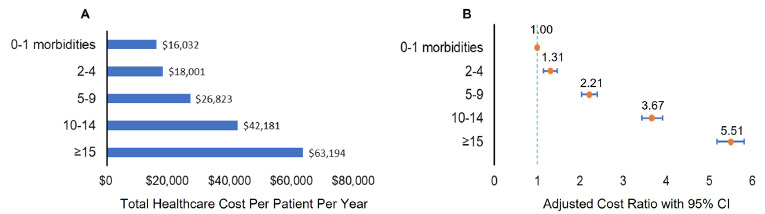
Total All-cause Healthcare Cost by Number of Comorbid Conditions Group (**A**) and Adjusted Cost Ratio of Comorbid Conditions Group (**B**) (**A**) Bar chart of total healthcare cost per patient per year by number of comorbid conditions group among patients with ASCVD. (**B**) Forest plot of multivariable adjusted cost ratios (ACR) of number of comorbid conditions group for total all-cause healthcare cost. Adjusted variables included age, gender, geographic region, rural or urban residence, household income, social vulnerability index, and type of health insurance. The *vertical dashed blue line* represents a ACR of 1 as the reference line, which is associated with equal costs for all number of comorbid conditions groups. For each ACR displayed, the *orange circle symbol* depicts the ACR, and the *horizontal blue line* represents the 95% CI. Lines that do not cross the reference line are statistically significant.

**Figure 3** presents the average total all-cause healthcare costs during the 12-month follow-up period after the index date by multimorbidity groups and multivariable adjusted cost ratios of multimorbidity for the total healthcare costs. Compared with patients without multimorbidity (0-1 morbidities), a higher multimorbidity burden was associated with higher healthcare costs in patients with ASCVD (all *P* < .0001). **Supplemental Figure S4** shows that the average healthcare costs increased with increases in the numbers of comorbid conditions among patients with ASCVD. The means (95% CI) of total healthcare costs were used to compare the differences among ASCVD patients with different numbers of comorbid conditions. After adjusting for other potential contributors to healthcare costs, (including age, gender, geographic region, rural or urban residence, household income, SVI, and type of health insurance), there was a 6.3% increase in total healthcare costs for patients with ASCVD for each additional comorbid condition (*P* < .0001).

## DISCUSSION

Most epidemiology studies on comorbidity and multimorbidity focus on limited number of individual comorbid conditions which co-present with ASCVD. Data on combinations of 2 or 3 comorbid conditions with ASCVD is limited, and data on 4 or more comorbid condition combinations is lacking.^3–8^ Information on the prevalence of individual comorbid conditions is useful but lacks the complexity that physicians and healthcare providers must navigate in routine clinical practice and disease management.^8^ Using a large US healthcare claims database, through association rule mining, we found a wide spectrum of comorbid conditions and their combinations among adult patients with ASCVD. The study demonstrates that 98.5% of patients with ASCVD had at least 2 comorbid conditions, 80.2% had at least 5 comorbid conditions, and 25.7% had at least 10 comorbid conditions. The mean number of comorbid conditions was 7 among patients with ASCVD. We also found that the multimorbidity burden tended to increase in older age groups and was comparatively higher in females than males, higher in patients with lower household income than with higher incomes, and higher in those with higher social vulnerability than in lower vulnerability. The most common comorbid condition dyad in ASCVD was hypertension-hyperlipidemia (78.7%). The most common triad was hypertension–hyperlipidemia–pain disorders (61.1%). The most common quartet was hypertension–hyperlipidemia–pain disorders–DM (30.2%). The most common quintet was hypertension–hyperlipidemia–pain disorders–DM–obesity (16%). The most common sextet was hypertension–hyperlipidemia–pain disorders–DM–obesity–osteoarthritis (7.6%). The prevalence of the most common comorbid conditions, condition dyads and triads are similar to the prevalence of those conditions among the patients with ischemic heart disease in the US Medicare population reported by CMS.^3,24^ To our knowledge, this study is the first to present the most prevalent 4 to 6 comorbid condition combinations in ASCVD.

ASCVD patients with multimorbidity frequently visit numerous medical generalists and specialists and utilize many healthcare resources to manage individual diseases, which may be inefficient, burdensome, and duplicative.^6^ Contemporary clinical practice guidelines usually provide diagnosis, therapeutics, and management strategies for a specific disease process but do not provide sufficient guidance for patients with multimorbidity. Providers usually adhere to standard of care clinical practice guidelines for individual chronic diseases, which may not be suitable when applied to the care of ASCVD patients with multimorbidity.^25^ Simply adding the recommended therapies for each comorbid condition leads to burdensome monitoring regimens and polypharmacy,^25,26^ increasing the risk for drug-drug interactions, drug-disease interactions, and therapeutic competition (in which recommended medications for one condition adversely affect other comorbid conditions), treatment complexity, and therapeutic confusion, especially in older patients.^2,6,25^ Comorbid conditions such as advanced HF, anxiety, depression, arthritis, and Alzheimer’s disease/dementia impair patients’ physical and cognitive functioning and pose significant barriers to treatment adherence and lifestyle recommendations.^27^ Therefore, ASCVD patients with multimorbidity require significant coordination between multidisciplinary care teams, prioritization of therapies, and substantial healthcare resources. Although these patients may be eligible for multiple therapies, clinical guidance on how to best sequence treatments and when to considering adding, reducing dose, stopping, or switching to other medications remains unclear. Clinicians caring for these patients need comprehensive management strategies on how to mitigate ASCVD progression and risk of ACEs within the context of multimorbidity and multiple guidance-directed therapies.

A behavioral risk factor surveillance study demonstrated that female gender, low household income, being unemployed, and difficulty accessing health care were significantly associated with a higher burden of CV comorbidities, including hypertension, hyperlipidemia, DM, current cigarette smoking, and chronic kidney disease among patients with ASCVD.^28^ The current study further shows that the number of comorbid conditions including CV and non-CV comorbid conditions in ASCVD tended to increase in older age groups and was highest in females, in patients with lower household income, and in those with higher social vulnerability. A previous study demonstrated that lower socioeconomic status was associated with higher ASCVD risk.^29^ Jain et al reported that SVI was associated with prevalent CV comorbidities and ASCVD.^30^ The 2019 ACC/ AHA guidelines recommend using social determinants of health (SDOH) to inform optimal strategies for the prevention of ASCVD.^1^ The SDOH or social vulnerability status should also inform evaluation and management strategies for the secondary prevention care among patients with ASCVD and multimorbidity.

ASCVD remains a leading cause of morbidity, mortality, and disability worldwide. In spite of innovative treatments, through pharmacological and interventional approaches, ASCVD continues to progress, especially due to the rising prevalence of chronic conditions, such as dyslipidemia, DM, hypertension, and other well-established ASCVD risk factors, but also because of the cumulative comorbid condition burden and emerging risk-enhancing factors, such as chronic kidney disease, metabolic syndrome, and chronic inflammatory conditions.^1,2,11,31^ The European Society of Cardiology identifies 4 key risk factors for ASCVD^31^: hypertension, hyperlipidemia, DM, and smoking—all of which were present in 88.9%, 86%, 41.9%, and 19.4 of patients with ASCVD in this study. Further, their combinations were also highly prevalent: hypertension-hyperlipidemia (78.7%), hypertension-hyperlipidemia-DM (37.1%), and hypertension-hyperlipidemia-DM-smoking (7.5%) in the cohort. Despite the high prevalence of multimorbidity, most research remains single disease–focused, and information on the impact of multimorbidity on ACEs is still lacking. We found that an increased number of comorbid conditions, including CV and non-CV comorbid conditions, was significantly associated with increased ACEs (including ACS, AMI, unstable angina, stroke, PAD, coronary revascularization, HF, hospitalization for HF, and MACE) and increased all-cause healthcare costs in the current study. Multimorbidity is defined as the presence of at least 2 comorbid conditions within a patient,^15–19^ yet most epidemiology studies of multimorbidity are based on counts of selected individual comorbid conditions.^2,8^ However, measuring the impact of multimorbidity based on counts of individual comorbid conditions may miss important heterogeneity, because the specific comorbid condition combinations affect health outcomes, health-related quality of life, and healthcare resource utilization and costs.^32–35^ Further research is needed to evaluate the impact of specific comorbid condition combinations on ACEs, health economics, quality of life, and quality of care in patients with ASCVD.

### Strengths

This study has two strengths that warrant consideration. First, the large sample of 223 923 patients with ASCVD from a large US commercial insured and Medicare Advantage population in the study allowed us to estimate the prevalence of individual comorbid conditions, specific comorbid condition combinations, and multimorbidity and assess the associations between multimorbidity and ACEs and healthcare costs among patients with ASCVD. Second, the strength of unsupervised data mining is the discovery of meaningful patterns that could not be discovered from large databases through conventional statistical methods.^8^ Association rule mining allows for a comprehensive identification of comorbid condition combinations from a large database in a quick and computationally efficient way, while filtering out infrequent comorbid conditions and condition combinations.^8,35,36^ Association rule mining has been widely used in marketing and business analytics. Although some applications have been reported recently,^36–39^ association rule mining is still relatively uncommon in clinical, epidemiology, and health outcomes research. To our knowledge, this is the first study to combine administrative claims analysis with association rule mining to evaluate the multimorbidity burdens in ASCVD.

### Limitations

This study has several limitations in the administrative claims data analysis that could affect interpretation of the study results. First, since ASCVD, ACEs, and all comorbid conditions were identified using ICD-10-CM codes only, misclassification, coding errors, and underestimation or overestimation are possible. However, the approach is routinely used in healthcare claims and electric health records analyses.^5,8,34,38^ Second, there is no standard or operational definition for comorbid conditions,^8,16,19,40^ so we included 42 common comorbid conditions in the study, which doubles 20 selected conditions in some multimorbidity studies.^17,35^ The CCI includes only 19 chronic conditions.^20^ Although the number of comorbid conditions is adequate to assess overall multimorbidity, some rare chronic conditions that may be associated with ACEs may be missed in this study. Third, lifestyle factors (eg, diet, exercise, weight, exposures), body mass index, blood pressure, and laboratory data were lacking in the healthcare claims databases. Fourth, we do not know patients’ race and ethnicity, marital status, education level, and social support, all of which may be associated with ACEs. Fifth, mortality data are lacking in healthcare claims data, so we could not evaluate the impact of multimorbidity on CV-related and all-cause mortality among patients with ASCVD in this study. Sixth, the study reflects multimorbidity patterns among patients with ASCVD and its associations with ACEs and healthcare costs in a large US commercially insured and Medicare Advantage population; therefore, these findings may not be generalized to the overall population, as those with other types of health insurance or uninsured may have different characteristics.^9,15,41^

## CONCLUSIONS

Multimorbidity is extremely prevalent among adult patients with ASCVD. Multimorbidity patterns vary considerably across ASCVD patients and by age, gender, household income, and social vulnerability status. Multimorbidity is strongly associated with increased ACEs and increased healthcare costs. This study highlights the importance of preventing and managing multimorbidity in ASCVD. The findings from this study should provide clinicians, health policy makers, healthcare providers, and the medical research community a better understanding of the burden of multimorbidity and their impact among patients with ASCVD providing preliminary insights into the development of innovative prevention and management strategies to improve holistic patient care, mitigate the disease progression and ACEs, and reduce overall healthcare costs for ASCVD patients with multimorbidity.

### Disclosures

D.D., J.F., X.S., L.L., V.W.P., and A. B. were employees of CVS Health at the time the study was conducted.

### Presentation

This work was presented in part as a poster at ICPE 2023, the 39th International Conference on Pharmacoepidemiology & Therapeutic Risk Management, August 23-27, 2023 in Halifax, Nova Scotia, Canada. The abstract was published in *Pharmacoepidemiol Drug Saf*. 2023;32(suppl 1):257. doi:10.1002/pds.5687

## Supplementary Material

Online Supplementary Material
