# Supplementary material for: Multimorbidity in Atherosclerotic Cardiovascular Disease and Its Associations With Adverse Cardiovascular Events and Healthcare Costs: A Real-World Evidence Study
Source: J Health Econ Outcomes Res. 2024 Mar 22;11(1):75–85. doi: 10.36469/001c.94710 (PMC10961141; doi:10.36469/001c.94710)
Supplement: Online Supplementary Material [file jheor_2024_11_1_94710_221129.pdf]

Journal of Health Economics and Outcomes Research

Online Supplementary Material

Multimorbidity in Atherosclerotic Cardiovascular Disease and Its Associations with Adverse Cardiovascular Events and Healthcare Costs: A Real-World Evidence Study. *JHEOR*. 2024;11(1):75-85. doi:10.36469/jheor.2024.94710

Table S1. ICD-10-CM Codes for Comorbid Conditions

Table S2. ICD-10-CM Codes for Clinical Adverse Cardiovascular Events

Figure S1. Study Design and Time Frame

Figure S2. Patient Attrition

Figure S3. Prevalence of Clinical Adverse Cardiovascular Events in ASCVD Patients with Different Number of Comorbid Conditions

Figure S4. Healthcare Costs Among ASCVD Patients With Different Number of Comorbid Conditions

This supplementary material has been provided by the authors to give readers additional information about their work.

This is an open-access article distributed under the terms of the Creative Commons Attribution 4.0 International License (CCBY-4.0). View this license's legal deed at http://creativecommons.org/licenses/by/4.0 and legal code at http://creativecommons.org/licenses/by/4.0/legalcode for more information.

Table S1. ICD-10-CM Codes for Comorbid Conditions

Comorbidity	Abbreviation	ICD-10-CM Codes
Atrial fibrillation	AFF	I48.0–I48.2, I48.3, I48.4, I48.91
AIDS/HIV	AID	B20.x–B22.x, B24.x
Anxiety	ANX	F40.x F41.x
Asthma	AST	J45.x
Aortic valve stenosis	AVS	I35.0-I35.2
Bipolar disorder	BIP	F31.x
Cancer (malignancy)	CAN	C00.x–C97.x
Congenital heart disease	CHD	Q20.x–Q28.x
Congestive heart failure	CHF	I09.9, I11.0, I13.0, I13.2, I25.5, I42.0, I42.5–I42.9, I43.x, I50.x, P29.0
Cholelithiasis/cholecystitis	CHO	K80.x
Chronic kidney disease	CKD	N18.1–N18.6, N18.9
Chronic obstructive pulmonary disease	COP	J40.x-J44.x, J47.x, J98.2, J98.3
Inflammatory bowel disease	CRO	K50.x–K52.x
Chronic thyroid disorders	CTD	E00.x–E07.x
Dissociative and conversion disorders	DCD	F44.x
Alzheimer's disease/dementia	DEM	F00.x–F03.x, F05.x, G13.8, G30.x, G31.1, G31.83, G94.x, R41.81
Depression	DEP	F32.x, F33.x
Diabetes mellitus	DM	E10.x–E13.x
Diverticular disease	DTD	K57.x
Epilepsy	EPL	G40.x
Fatigue and sleep related disorders	FSR	R53.0, R53.1, R53.8x, F48.8, F51.02, F51.09, F51.01, F51.03-F51.05, F51.19, F51.11-F51.13, F51.8, G47.0x, G47.1x, G47.2x, G47.4x-G47.6x, G47.8, G47.9, G93.3
Hypercoagulable syndrome	HCG	D68.59, D68.69
Paralysis (hemiplegia or paraplegia)	HEM	G04.1, G11.4, G80.1, G80.2, G81.x, G82.x, G83.0-G83.4, G83.9
Hyperlipidaemia	HLD	E78.0-E78.5
Hypertension	HTN	I10.x–I13.x, I15.x
Iron deficiency anemia	IDA	D50.8, D50.9
Kidney stones	KST	N20.x
Liver disease	MSL	B18.x, I85.0, I85.9, I86.4, I98.2, K70.0-K70.4, K70.9, K71.1, K71.3-K71.5, K71.7, K72.1, K72.9, K73.x, K74.x, K76.0, K76.2-K76.9, Z94.4
Multiple sclerosis	MSS	G35.x
Obesity	OBE	E66.x, Z68.3x, Z68.4x
Other nontoxic goiter	ONG	E04
Osteoporosis	OSP	M80.x, M81.x
Osteoarthritis	OST	M15.x–M19.x
Pain disorders	PAI	E08.42, E09.42, E11.42, E13.42, G04.xx, G05.xx, G35, G36.xx, G37.xx, G43.xx, G44.xx, G50.xx, G51.xx, G54.xx, G56.xx, G57.xx, G58.7, G60.xx, G89.xx, G90.xx, G95.xx, G99.xx, M05.xx, M06.xx, M12.xx, M26.6xx, M43.xx, M45.xx, M46.xx, M47.xx, M48.xx, M50.xx, M51.xx, M53.xx, M54.xx, M60.xx, M79.xx, M96.1, R07.xx, R10.xx, R16.xx, R19.xx, R51, R52, F45.4x, B02.2x, S04.1x, S14.1x, S24.1x, S34.1x, S34.3x, S44.xx, S54.xx, S64.xx, or S74.xx
Pancreatitis	PAN	K85.x
Parkinson's disease	PAR	G20.x
Psoriasis	PSO	L40.x
Psychoses	PSY	F20.x, F22.x–F25.x, F28.x, F29.x, F30.2
Rheumatoid arthritis/collagen vascular disease	RHA	L94.0, L94.1, L94.3, M05.x, M06.x, M08.x, M12.0, M12.3, M30.x, M31.0–M31.3, M32.x–M35.x, M45.x, M46.1, M46.8, M46.9

Table S1. ICD-10-CM Codes for Comorbid Conditions, *cont'd*

Comorbidity	Abbreviation	ICD-10-CM Codes
Stress and adjustment disorders	SAD	F43.x
Schizophrenia	SCH	F20.x
Substance use disorders	SDO	F10.x-F19.x
Systemic lupus erythematosus	SLE	M32.x
Ventricular arrhythmia	VNA	I47.x, I49.0x, I49.3x

Abbreviations: ICD-10-CM, *International Classification of Diseases, Tenth Revision, Clinical Modification*.

Table S2. ICD-10-CM Codes for Clinical Adverse Cardiovascular Events

Adverse Cardiovascular Events	Abbreviation	ICD-10-CM Codes
Acute coronary syndrome/ischemic heart disease	ACS	I21.x–I24.x
Acute myocardial infarction	AMI	I21.x, I22.x
Unstable angina	UA	I20.0, I23.7, I25.110, I25.700, I25.710, I25.720, I25.730, I25.750, I25.760, I25.790
Heart failure	HF	I11.0, I13.0, I13.2, I50.x, I90.81, I97.1
Peripheral artery disease	PAD	E08.51, E08.52, E10.51, E10.52, E11.51, E11.52, E13.51, E13.52, I70, I71
Stroke	STR	I63.x, I69.3
Coronary revascularization	CR	T82.21, Z95.1, Z95.5, Z95.818, Z95.9, Z98.6, Z98.61

Abbreviations: ACEs, adverse cardiovascular events; ICD-10-CM, *International Classification of Diseases, Tenth Revision, Clinical Modification.*

Figure S1. Study Design and Time Frame

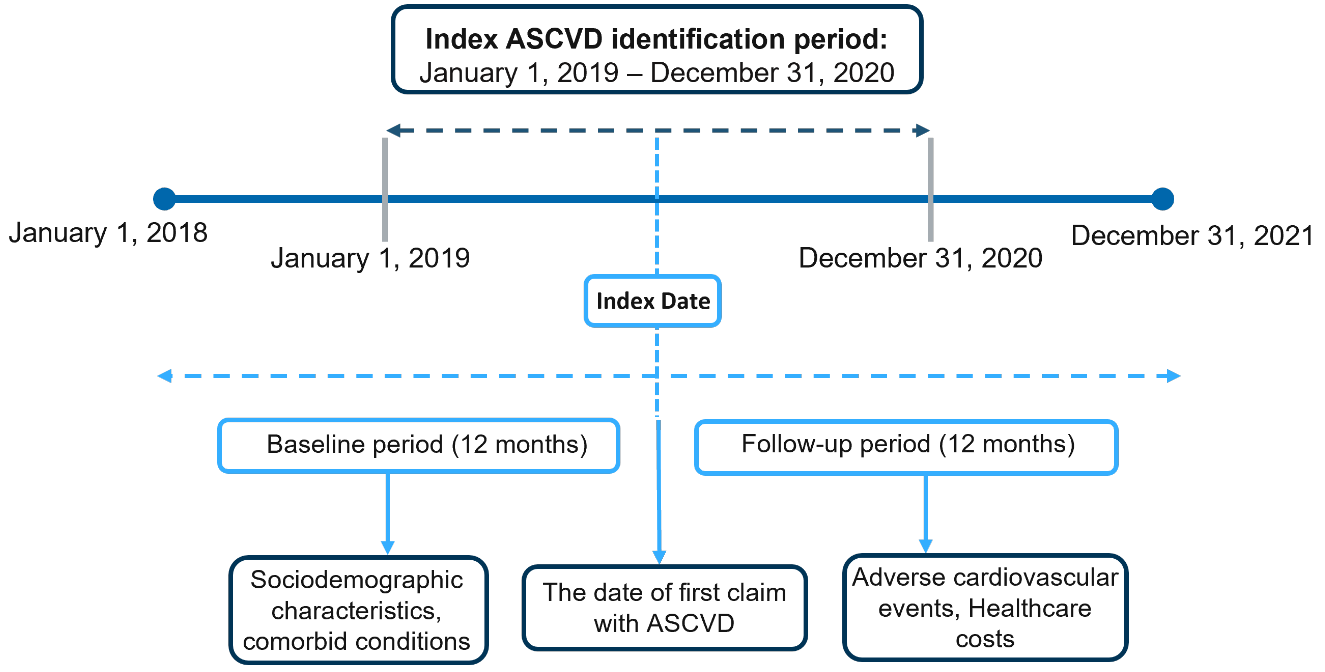

Figure S2. Patient Attrition

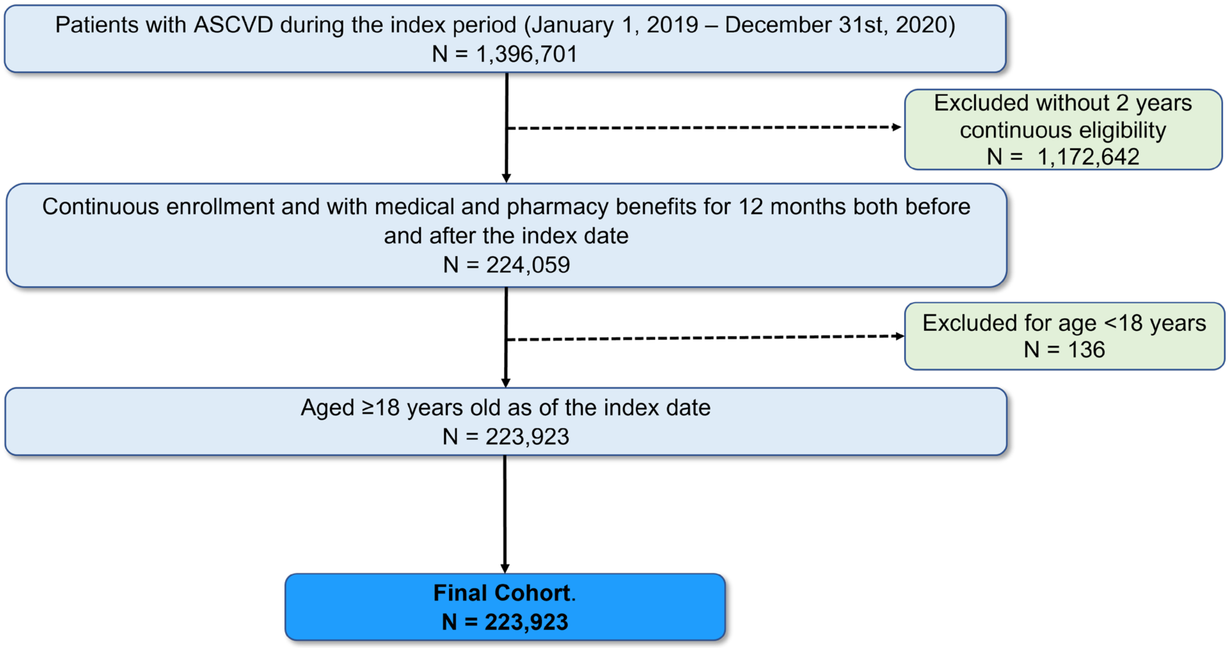

Figure S3. Prevalence of Clinical Adverse Cardiovascular Events in ASCVD Patients with Different Number of Comorbid Conditions

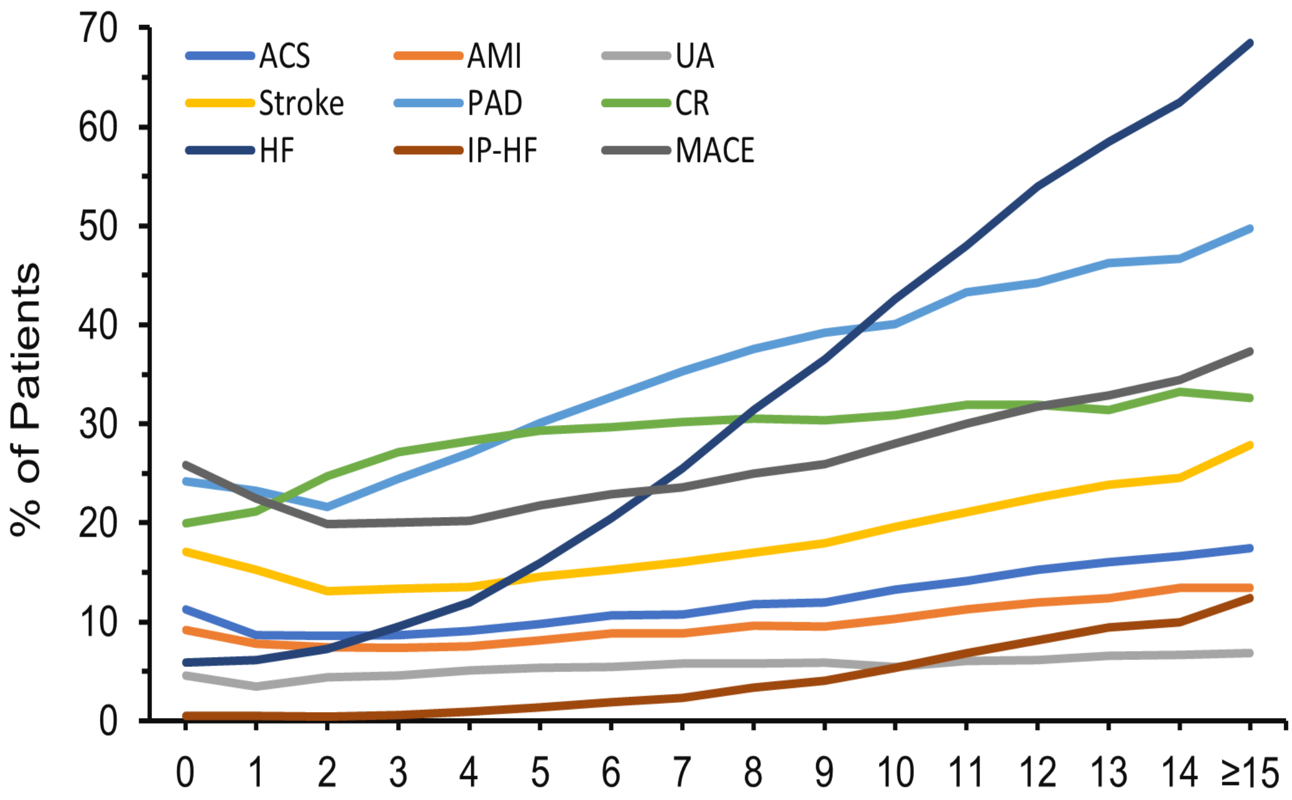

Abbreviations: ACEs, adverse cardiovascular events; ACS, acute coronary syndrome/ischemic heart disease; AMI, acute myocardial infarction; CR, coronary revascularization; HF, heart failure; IP-HF, heart failure related hospitalization; MACE, major adverse cardiovascular events; PAD, peripheral artery disease.

Figure S4. Healthcare Costs Among ASCVD Patients With Different Number of Comorbid Conditions

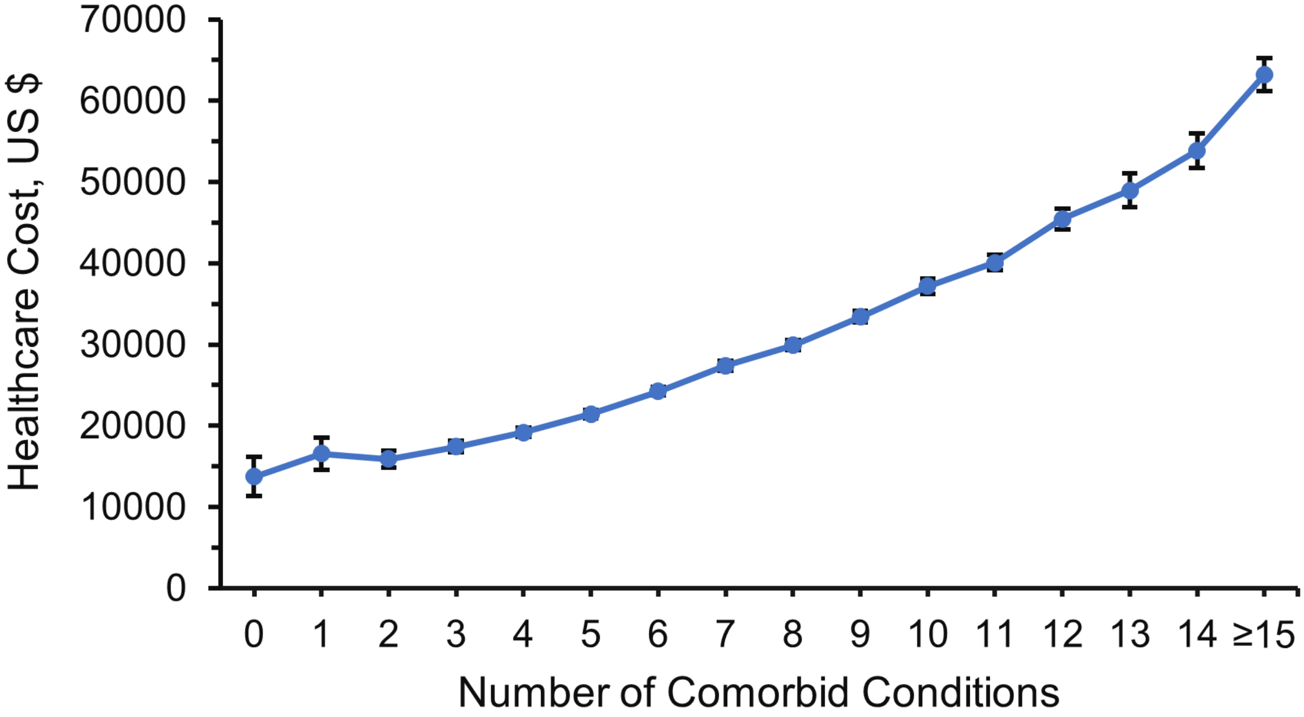

Solid dots indicate the means, and the *high and low bars* indicate 95% confidence intervals.
